# HADS‐Assessed Anxiety/Depression Status and Ocular Outcomes in Vision‐Threatening Diabetic Retinopathy: A Multicenter Retrospective Cohort Study

**DOI:** 10.1155/da/6998782

**Published:** 2026-10-25

**Authors:** Bo Li, Yujin Jiang, Chenxin Li, Yikeng Huang, Shuzhi Zhao, Chuandi Zhou, Zhi Zheng, Xiaofeng Zhang

**Affiliations:** ^1^ Department of Ophthalmology The Fourth Affiliated Hospital of Soochow University No. 9 Chongwen Road, Suzhou Industrial Park Suzhou 215123 Jiangsu Province China; ^2^ Department of Ophthalmology Shanghai General Hospital National Clinical Research Center for Eye Diseases Shanghai Jiao Tong University School of Medicine No.100 Haining Road, Hongkou District Shanghai 200080 China shsmu.edu.cn; ^3^ Department of Ophthalmology Ninth People’s Hospital Shanghai Jiao Tong University School of Medicine No. 639 Zhizaoju Road, Huangpu District Shanghai 200011 China shsmu.edu.cn

**Keywords:** anxiety, depression, ocular outcomes, vision-threatening diabetic retinopathy

## Abstract

**Purpose:**

Vision‐threatening diabetic retinopathy (VTDR) poses significant blindness risks and the study aimed to identify the impact of psychological factors on its ocular outcomes.

**Methods:**

This retrospective cohort study enrolled 782 VTDR patients from three ophthalmological centers (2018–2021). DR severity was graded using ETDRS criteria. Primary outcomes included tractional retinal detachment (TRD) involving the fovea, neovascular glaucoma (NVG), final best‐corrected visual acuity (BCVA) <0.3, and recurrent vitreous hemorrhage (VH). Anxiety and depression were assessed via the hospital anxiety and depression scale (HADS). Univariable and multivariable logistic regression evaluated the associations between HADS scores and ocular outcomes.

**Results:**

After a median follow‐up of 24.8 months, depressive symptoms (HADS‐D ≥8) were associated with higher rates of BCVA <0.3 (67.8% vs. 57.2%, *p* = 0.033) and NVG (13.6% vs. 6.9%, *p* = 0.020). Recurrent VH was more frequent in patients with anxiety (21.0% vs. 10.6%, *p* = 0.031) or depression (20.3% vs. 10.7%, *p* = 0.045). Multivariable logistic analysis revealed that each 1‐point increase in HADS‐D score significantly increased adjusted odds of TRD (aOR = 1.06, 95% CI: 1.01−1.11), BCVA <0.3 (aOR = 1.07, 95% CI: 1.02−1.11), recurrent VH (aOR = 1.09, 95% CI: 1.02−1.17), and NVG (aOR = 1.10, 95% CI: 1.03−1.17; all *p* < 0.05). There was a consistent positive gradient between HADS severity and adverse outcomes.

**Conclusion:**

Depressive symptoms are independently associated with worse ocular prognoses in VTDR patients. Systematic psychological screening and intervention is essential to mitigate vision deterioration risk in this population.

## 1. Introduction

Diabetic retinopathy (DR), a severe microvascular ocular complication of diabetes, is increasing in prevalence alongside the rising global burden of diabetes. It is estimated that ~103 million people worldwide have been diagnosed with DR, constituting 22.3% of the global diabetic population, with this number projected to reach 161 million by 2045 [1]. Based on the stages of retinopathy, DR is classified into nonproliferative diabetic retinopathy (NPDR) and proliferative diabetic retinopathy (PDR) [2]. Severe NPDR, PDR, and diabetic macular edema (DME) are collectively termed vision‐threatening diabetic retinopathy (VTDR) [3]. Generally, pan‐retinal photocoagulation (PRP) and vitrectomy are considered the established interventions to halt disease progression for severe NPDR and PDR. Anti‐vascular endothelial growth factor (anti‐VEGF) therapy is regarded as the first‐line treatment for DME [4]. However, patients undergoing ocular surgery usually face significant physical trauma, whereas anti‐VEGF therapy is challenged by suboptimal response, substantial financial burden, and poor treatment adherence [5]. Although the global prevalence of VTDR is only 6.7%, this burden is compounded by the conflict between limited sustainable treatment strategies and the necessity for long‐term management, leading to exacerbated visual impairment and a significant societal burden [6]. Consequently, early prevention of DR is critically important.

Multiple risk factors have been demonstrated to promote the development and progression of DR, including long diabetes duration, uncontrolled metabolic risk factors (glycated hemoglobin [HbA1c], blood glucose, blood pressure, and lipids), unhealthy lifestyle factors (smoking, alcohol abuse, poor diet quality, physical inactivity, and sleep disorders), and lower socioeconomic status (less educational level, economic disadvantage, and unemployment) [7–9]. Additionally, psychological factors have also been reported as significant risk factors for DR onset and progression. Evidence has indicated that individuals with DR experienced lower quality of life and poorer psychosocial outcomes [10]. Furthermore, chronic depression and anxiety in these patients are associated with significantly reduced treatment adherence and self‐management capabilities, thereby exacerbating DR progression and creating a vicious cycle [11]. This evidence suggests a potential bidirectional relationship between adverse psychological status and DR. However, the association between adverse psychological symptoms and ocular outcomes, specifically in VTDR patients, remains unexplored.

In this study, we enrolled 782 VTDR patients from three ophthalmic centers to investigate the relationship between anxiety and depression symptoms (assessed using the HADS) and post‐treatment ocular outcomes following standard therapy.

## 2. Methods

### 2.1. Participants and Ethics Approval

From January 2018 to December 2021, patients newly diagnosed with VTDR in the Department of Ophthalmology, Shanghai General Hospital, the Ninth People’s Hospital affiliated to Shanghai Jiao Tong University School of Medicine, and the Fourth Affiliated Hospital of Soochow University were enrolled. Participants with any of the following conditions were excluded: (1) follow‐up period <1 year; (2) inaccessibility to standardized treatment; (3) severe visual impairment from non‐DR etiologies; (4) missing mental status or VTDR diagnostic data in medical records; and (5) suffering from major psychological trauma resulted in an alteration of the assessment findings. The study was approved by the Institutional Review Board of Shanghai General Hospital (ID: 2022KY024). And the requirement for informed consent was waived owing to the retrospective design of the study. The tenets of the Declaration of Helsinki was adhered to.

### 2.2. Main Ocular Outcomes and Mental Status Definitions

The medical records of enrolled patients with DR were reviewed, and the severity of DR was graded based on the early treatment diabetic retinopathy study (ETDRS) grading standards [12]. Specifically, VTDR was defined as severe NPDR, PDR, and clinically significant DME. Optical coherence tomography (OCT) was employed to confirm clinically significant DME, defined as retinal edema or fovea‐involving hard exudates. Participants received one or more of the following standardized treatments for VTDR, such as PRP for severe NPDR and PDR, intravitreal injections of anti‐VEGF agents for DME or those with active fibrovascular proliferation in eyes scheduled for vitrectomy, intravitreal injections of dexamethasone implant for refractory DME, and pars plana vitrectomy (PPV) for vitreous hemorrhage (VH) and tractional retinal detachment (TRD), as formerly described [13].

Four major clinical outcomes were defined as the presence of TRD involving the fovea, neovascular glaucoma (NVG), final best‐corrected visual acuity (BCVA) <0.3 (the better eye, consistent with the World Health Organization’s low vision criteria), and recurrent VH post‐vitrectomy. All outcomes were assessed until the follow‐up ended. TRD involving the fovea and recurrent VH were definitively diagnosed by board‐certified ophthalmologists through indirect ophthalmoscopy under dilated pupil conditions, with subsequent confirmation via OCT and ocular B‐scan ultrasonography. NVG was diagnosed by slit‐lamp biomicroscopy and gonioscopy [14]. BCVA was tested employing a Snellen chart, converted to the logarithm of the minimum angle of resolution (logMAR), and recorded using decimal notation. Participants’ ophthalmic medical records were reviewed and assessed independently by two qualified ophthalmologists. Any discrepancy was resolved by a third evaluation from a senior ophthalmologist, and the definitive assessment was recorded.

The hospital anxiety and depression scale (HADS) was utilized to assess the symptoms of depression and anxiety among patients diagnosed with VTDR. Briefly, the HADS is a 14‐item psychometric instrument comprising two discrete 7‐item subscales quantifying anxiety and depressive symptomatology. Each item is scored on a 4‐point Likert scale (0–3 score), with cumulative subscale scores ranging from 0 to 21. A validated clinical cutoff score of ≥8 on either subscale indicates anxiety or depressive symptoms, with a score of 8–10 classified as borderline and >10 as clinically significant [15–17]. The HADS questionnaires were completed as a self‐report by patients before receiving the standard treatment, using standardized instructions delivered by trained research assistants. Completed forms were checked for missing responses, and all data were independently entered and cross‐verified by two researchers.

### 2.3. Covariate Definitions

The baseline characteristics were systematically documented on the day of VTDR diagnosis. Demographic characteristics encompassed age, gender (male or female), occupational status (occupied or unoccupied), educational attainment (less than college or college school and higher), marital status (married, unmarried, or other), and self‐reported perceived financial burden (slight, moderate, or heavy). Key clinical parameters, including diabetes type (type 1 or 2 diabetes), diabetic duration, body mass index (BMI), systolic and diastolic blood pressure (SBP and DBP), HbA1c, low‐density lipoprotein cholesterol (LDL), and diabetic nephropathy (DN) were systematically documented using standardized protocols. DN was defined as estimated glomerular filtration rate (eGFR) <60 mL/min/1.73 m^2^ or urinary albumin to creatinine ratio (ACR) ≥30 mg/g. Cardiovascular disease (CVD) was defined as a history of any congestive heart failure, coronary heart disease, myocardial infarction, or stroke, as documented in electronic medical records or self‐reported by patients. Additionally, smoking status was also documented (current or former smoker and nonsmoker). For participants screened as having borderline or clinical anxiety and depression, their usage of antidepressant and anti‐anxiety medications was recorded and defined as either use or non‐use.

### 2.4. Sample Size Calculation

Sample size estimation was grounded in the prevalence of anxiety and depression reported for patients with DR in prior studies [18, 19]. Given that our investigation specifically concerns patients with VTDR, who are expected to face a heightened burden of psychological distress relative to the conventional DR population, we conservatively estimated the prevalence of anxiety/depression in this subgroup at 40%. The minimum sample size required for estimating a single proportion was determined using the formula: *n* = *Z* ^2^_(1–*α*/2)_ × *p* ^∗^(1–*p*)/*d* ^2^, setting *Z*_(1–*α*/2)_ = 1.96 (two‐sided 95% confidence level), *p* = 0.40 (based on the literature‐derived estimate), and *d* = 0.05 (allowable margin of error). This calculation produced a required sample size of ~369 patients for the VTDR cohort. Accounting for an expected 20% rate of incomplete responses or loss to follow‐up, we aimed to recruit 462 VTDR patients. In practice, a total of 782 participants were enrolled, surpassing the calculated minimum and thus ensuring robust statistical power. All sample size computations were executed using PASS software (version 21.0, NCSS, LLC, Kaysville, UT, USA).

### 2.5. Statistical Analysis

The distribution of demographic and clinical characteristics was assessed and proper statistical methods were employed to compare participants with different mental status, using the Student’s *t*‐test, the nonparametric Mann–Whitney *U* test, the chi‐square test, or Fisher’s exact test as appropriate. Additionally, univariate and multivariate logistic regression models were utilized to identify the association between anxiety or depression and ocular outcomes among VTDR patients. *P* for trend was calculated by assigning median scores to each group and treating the variable as continuous in logistic regression. All statistical analyses were conducted using IBM SPSS Statistics 25.0 (SPSS, Inc., Chicago, IL). A two‐sided *p*‐value <0.05 was considered significant.

## 3. Results

### 3.1. Descriptive Statistics

The study cohort comprised 782 participants diagnosed with VTDR, predominantly male (58.8%) and with a median age of 57.0 years (IQR: 48.0–64.0). The vast majority had type 2 diabetes (94.4%) and the median diabetes duration was 15.0 years (IQR: 10.0–20.0). Most participants had less than college education (92.1%), were married (92.1%), and experienced moderate (56.8%) or heavy (34.8%) economic burden, with 63.7% being unoccupied and 32.7% having smoking habits. For metabolic factors, median HbA1c was 7.5% (IQR: 6.9–8.1), systolic blood pressure 133.0 mmHg (IQR: 123.0–145.0), DBP 80.0 mmHg (IQR: 74.0–87.0), LDL cholesterol 2.7 mmol/L (IQR: 2.1–3.2), and BMI 24.0 kg/m^2^ (IQR: 22.0–26.0). The prevalence of DN was 61.6% (*N* = 482) in the population. Regarding the mental status, 15.6% and 15.1% of participants had anxiety (borderline: 7.9% and clinical: 7.7%) and depression (borderline: 10.0% and clinical: 5.1%) symptoms identified by the HADS (median: 6, IQR: 3–11). Among participants screened for borderline or clinical anxiety, 8 individuals (8/122, 6.6%) used anxiolytic medications, while for depression screening, this figure was 10 (10/118, 8.5%). The characteristics of the enrolled patients are shown in Table 1.

**Table 1:** Demographics and baseline clinical characteristics.

Variables	No. (%) or median (IQR) (*N* = 782)
Age, median (IQR), year	57.0 (48.0,64.0)
Gender, no. (%)	
Male	460 (58.8)
Female	322 (41.2)
Duration of diabetes, median (IQR), year	15.0 (10.0,20.0)
Type of diabetes, no. (%)	
Type 1 diabetes	44 (5.6)
Type 2 diabetes	738 (94.4)
Educational level, no. (%)	
Less than college school	720 (92.1)
College school and higher	62 (7.9)
Marital status, no. (%)	
Married	720 (92.1)
Unmarried	46 (5.9)
Others	16 (2.0)
Self‐reported economic burden, no. (%)	
Slight	66 (8.4)
Moderate	444 (56.8)
Heavy	272 (34.8)
Occupational status, no. (%)	
Occupied	284 (36.3)
Unoccupied	498 (63.7)
HbA1c, median (IQR), %	7.5 (6.9,8.1)
SBP, median (IQR), mmHg	133.0 (123.0,145.0)
DBP, median (IQR), mmHg	80.0 (74.0,87.0)
LDL, median (IQR), mmol/L	2.7 (2.1,3.2)
BMI, median (IQR), kg/m^2^	24.0 (22.0,26.0)
HADS score, median (IQR)	6.0 (3.0,11.0)
Anxiety status, no. (%)	
Without anxiety	660 (84.4)
Borderline anxiety	62 (7.9)
Clinical anxiety	60 (7.7)
Anti‐anxiety medications usage	8 (6.6)
Depression status, no. (%)	
Without depression	664 (84.9)
Borderline depression	78 (10.0)
Clinical depression	40 (5.1)
Anti‐depressant medications usage	10 (8.5)
DN, no. (%)	
With DN	482 (61.6)
Without DN	300 (38.4)
CVD, no. (%)	
With CVD	166 (21.2)
Without CVD	616 (78.8)
Smoking status, no. (%)	
Current or former smoker	256 (32.7)
No smoker	526 (67.3)

*Note:* The usage of anti‐anxiety/deprssion medications was only obtained from those participants with borderline or clinical anxiety/depression.

Abbrevations: BMI, body mass index; CVD, cardiovascular disease; DBP, diastolic blood pressure; DN, diabetic nephropathy; HbA1c, glycated hemoglobin; IQR, interquartile range; LDL, low density lipoprotein; SBP, systolic blood pressure.

### 3.2. The Prevalence of Ocular Outcomes Among Participants With Different Mental Statuses

After a median follow‐up of 24.8 months, the analysis revealed significant associations between the psychological status and specific ocular outcomes for VTDR (*N* = 782, shown in Table 2). Patients with depression (HADS‐Depression subscore ranging from 8 to 21) exhibited a significantly higher rate of final BCVA <0.3 (67.8% vs. 57.2%, *p* = 0.033) and a higher incidence of NVG (13.6% vs. 6.9%, *p* = 0.020) compared to those without depression. Recurrent VH among patients undergoing PPV (*N* = 381) was significantly more frequent in both patients with anxiety (HADS‐Anxiety subscore ranging from 8 to 21) (21.0% vs. 10.6%, *p* = 0.031) and patients with depression (20.3% vs. 10.7%, *p* = 0.045). No statistically significant differences were observed based on anxiety or depression status for TRD involving the fovea, nor for the overall rate of NVG when stratified by anxiety status. However, as described in Figure 1, stratified analysis by severity of psychological symptoms revealed a consistent positive relationship between HADS subscale scores and adverse ophthalmic outcomes (all *p* for trend <0.05).

**Figure 1:**
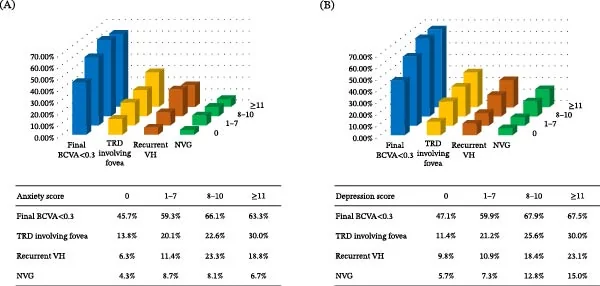
The prevalence of main ocular outcomes among different anxiety/depression score groups. (A) The prevalence of final BCVA <0.3, TRD involving foveal, recurrent VH and NVG among different anxiety score groups (0, 1–7, 8–10, and ≥11). (B) The prevalence of final BCVA <0.3, TRD involving foveal, recurrent VH and NVG among different depression score groups (0, 1–7, 8–10, and ≥11). BCVA, best‐corrected visual acuity; NVG, neovascular glaucoma; TRD, tractional retinal detachment; VH, vitreous hemorrhage.

**Table 2:** Clinical outcomes after at least 1‐year of standardized treatments for VTDR.

HADS score	Anxiety status		*p*‐Value	Depression status		*p*‐Value
	0–7	8–21		0–7	8–21	
TRD involving foveal (*n* = 782)	127 (19.2)	32 (26.2)	0.087	127 (19.1)	32 (27.1)	0.062
Final BCVA <0.3 (*n* = 782)	381 (57.7)	79 (64.8)	0.161	380 (57.2)	80 (67.8)	0.033 ^∗^
Recurrent VH (*n* = 381)	34 (10.6)	13 (21.0)	0.031 ^∗^	34 (10.7)	13 (20.3)	0.045 ^∗^
NVG (*n* = 782)	53 (8.0)	9 (7.4)	0.821	46 (6.9)	16 (13.6)	0.020 ^∗^

*Note:* Data presented are number (%). Recurrent VH was only counted in patients underwent pars plana vitrectomy.

Abbreviations: BCVA, best‐corrected visual acuity; NVG, neovascular glaucoma; TRD, tractional retinal detachment; VH, vitreous hemorrhage; VTDR, vision‐threatening diabetic retinopathy.

^∗^Statistically significant.

### 3.3. The Association Between Ocular Outcomes and Mental Status

Further, univariable and multivariable logistic regression was employed to identify the relationship between the specific ocular complications and HADS score‐based mental symptoms (Tables 3 and 4). Unadjusted analysis revealed significant associations between higher HADS scores and increased odds of adverse ocular outcomes, with depression demonstrating broader effects than anxiety. Following adjustment for systemic confounders, including personal characteristics, diabetic indicators, metabolic factors, medication usage, and socioeconomic status, depression showed robust independent associations: each 1‐point increase in HADS‐Depression score was associated with increased adjusted odds of TRD involving the fovea (aOR = 1.06, 95% CI: 1.01–1.12, *p* = 0.015), final BCVA <0.3 (aOR = 1.05, 95% CI: 1.01–1.10, *p* = 0.022), recurrent VH (aOR = 1.09, 95% CI: 1.01–1.17, *p* = 0.049), and NVG (aOR = 1.09, 95% CI: 1.01–1.17, *p* = 0.027). Clinically significant depression (HADS ≥8) was independently associated with the risk of NVG (aOR = 2.33, 95% CI: 1.20–4.55, *p* = 0.013), and severe depression (scores 11–21) was markedly associated with NVG incidence (aOR = 2.97, 95% CI: 1.07–8.23, *p* = 0.011). In contrast, most associations for anxiety were attenuated after adjustment, retaining significance only for the per‐point increase in final BCVA <0.3 (aOR = 1.04, 95% CI: 1.01–1.09, *p* = 0.049).

**Table 3:** The association of HADS score and clinical characteristics of VTDR in unadjusted models.

Ocular outcomes	HADS score	Anxiety status			Depression status		
		No. (%)	OR (95% CI)	*p*‐Value	No. (%)	OR (95% CI)	*p*‐Value
TRD involving foveal	0–7	127 (19.2)	Ref	—	127 (19.1)	Ref	—
	8–10	14 (22.6)	1.22 (0.66,2.29)	0.527	20 (25.6)	1.46 (0.85,2.51)	0.174
	11–21	18 (30.0)	1.80 (1.01,3.23)	0.048 ^∗^	12 (30.0)	1.81 (0.90,3.66)	0.098
	For trend	—	1.32 (1.01,1.74)	0.047 ^∗^	—	1.38 (1.01,1.87)	0.042 ^∗^
	Per 1 score increase	—	1.05 (1.01,1.10)	0.049 ^∗^	—	1.08 (1.03,1.13)	0.001 ^∗^
	No symptom	127 (19.2)	Ref	—	127 (19.1)	Ref	—
	Existing symptom	32 (26.2)	1.49 (0.95,2.33)	0.080	32 (27.1)	1.57 (1.01,2.47)	0.048 ^∗^

Final BCVA <0.3	0–7	381 (57.7)	Ref	—	380 (57.2)	Ref	—
	8–10	41 (66.1)	1.43 (0.83,2.47)	0.201	53 (67.9)	1.58 (0.96,2.61)	0.071
	11–21	38 (63.3)	1.27 (0.73,2.19)	0.400	27 (67.5)	1.55 (0.79,3.06)	0.205
	For trend	—	1.18 (0.91,1.52)	0.211	—	1.34 (1.01,1.80)	0.048 ^∗^
	Per 1 score increase	—	1.05 (1.01,1.10)	0.011 ^∗^	—	1.07 (1.03,1.11)	0.002 ^∗^
	No symptom	381 (57.7)	Ref	—	380 (57.2)	Ref	—
	Existing symptom	79 (64.8)	1.35 (0.90,2.01)	0.148	80 (67.8)	1.57 (0.14,2.38)	0.033 ^∗^

Recurrent VH	0–7	34 (5.2)	Ref	—	34 (5.1)	Ref	—
	8–10	7 (11.3)	2.34 (1.00,5.53)	0.050 ^∗^	7 (9.0)	1.83 (0.78,4.27)	0.165
	11–21	6 (10.0)	2.05 (0.82,5.09)	0.124	6 (15.0)	3.27 (1.29,8.32)	0.013 ^∗^
	For trend	—	1.54 (1.02,2.32)	0.039 ^∗^	—	1.81 (1.18,2.79)	0.007 ^∗^
	Per 1 score increase	—	1.08 (1.01,1.16)	0.024 ^∗^	—	1.12 (1.05,1.19)	0.001 ^∗^
	No symptom	34 (5.2)	Ref	—	34 (5.1)	Ref	—
	Existing symptom	13 (10.7)	2.20 (1.12,4.29)	0.022 ^∗^	13 (11.0)	2.29 (1.17,4.49)	0.015 ^∗^

NVG	0–7	53 (8.0)	Ref	—	46 (6.9)	Ref	—
	8–10	5 (8.1)	1.01 (0.39,2.61)	0.992	10 (12.8)	1.98 (0.95,4.10)	0.067
	11–21	4 (6.7)	0.82 (0.29,2.34)	0.708	6 (15.0)	2.37 (0.95,5.94)	0.065
	For trend	—	0.92 (0.58,1.48)	0.743	—	1.63 (1.09,2.44)	0.017 ^∗^
	Per 1 score increase	—	1.00 (0.93,1.07)	0.967	—	1.08 (1.01,1.14)	0.019 ^∗^
	No symptom	53 (8.0)	Ref	—	46 (6.9)	Ref	—
	Existing symptom	9 (7.4)	0.91 (0.44,1.90)	0.806	16 (13.6)	2.11 (1.15,3.86)	0.016 ^∗^

Abbrevations: BCVA, best‐corrected visual acuity; CI, confidence interval; NVG, neovascular glaucoma; OR, odds ratios; TRD, tractional retinal detachment; VH, vitreous hemorrhage; VTDR, vision‐threatening diabetic retinopathy.

^∗^Statistically significant.

**Table 4:** The impact of HADS score on VTDR adjusted for systemic parameters^†^

Ocular outcomes	HADS score	Anxiety status		Depression status	
		OR (95% CI)	*p*‐Value	OR (95% CI)	*p*‐Value
TRD involving foveal	0–7	Ref	—	Ref	—
	8–10	1.09 (0.56,2.12)	0.793	1.35 (0.76,2.42)	0.308
	11–21	1.44 (0.75,2.74)	0.275	1.35 (0.61,2.96)	0.456
	For trend	1.18 (0.87,1.60)	0.286	1.21 (0.86,1.71)	0.267
	Per 1 score increase	1.02 (0.97,1.08)	0.351	1.06 (1.01,1.12)	0.015 ^∗^
	No symptom	Ref	—	Ref	—
	Existing symptom	1.25 (0.77,2.04)	0.365	1.35 (0.83,2.21)	0.230

Final BCVA <0.3	0–7	Ref	—	Ref	—
	8–10	1.39 (0.79,2.45)	0.259	1.49 (0.88,2.50)	0.136
	11–21	1.17 (0.65,2.09)	0.607	1.24 (0.61,2.54)	0.552
	For trend	1.13 (0.87,1.48)	0.364	1.22 (0.90,1.67)	0.203
	Per 1 score increase	1.04 (1.01,1.09)	0.049 ^∗^	1.05 (1.01,1.10)	0.022 ^∗^
	No symptom	Ref	—	Ref	—
	Existing symptom	1.28 (0.84,1.95)	0.259	1.40 (0.91,2.17)	0.129

Recurrent VH	0–7	Ref	—	Ref	—
	8–10	3.17 (1.11,9.11)	0.032	1.64 (0.61,4.40)	0.325
	11–21	1.50 (0.50,4.56)	0.473	1.68 (0.52,5.45)	0.389
	For trend	1.41 (0.85,2.33)	0.181	1.36 (0.80,2.33)	0.257
	Per 1 score increase	1.06 (0.97,1.15)	0.185	1.09 (1.01,1.17)	0.049 ^∗^
	No symptom	Ref	—	Ref	—
	Existing symptom	2.18 (0.96,4.94)	0.064	1.66 (0.73,3.74)	0.225

NVG	0–7	Ref	—	Ref	—
	8–10	1.06 (0.39,2.88)	0.904	2.07 (0.94,4.56)	0.072
	11–21	0.85 (0.28,2.62)	0.777	2.97 (1.07,8.23)	0.036 ^∗^
	For trend	0.95 (0.58,1.57)	0.842	1.80 (1.15,2.84)	0.011 ^∗^
	Per 1 score increase	0.99 (0.92,1.08)	0.843	1.09 (1.01,1.17)	0.027 ^∗^
	No symptom	Ref	—	Ref	—
	Existing symptom	0.96 (0.44,2.09)	0.920	2.33 (1.20,4.55)	0.013 ^∗^

Abbrevations: BCVA, best‐corrected visual acuity; CI, confidence interval; NVG, neovascular glaucoma; OR, odds ratios; TRD, tractional retinal detachment; VH, vitreous hemorrhage; VTDR, vision‐threatening diabetic retinopathy.

^∗^Statistically significant.

†Adjusted systemic parameters included age, gender, educational level, marital status, self‐reported economic burden, occupational status, smoking, type of diabetes, duration of diabetes, HbA1c, low density lipoprotein, systolic blood pressure, body mass index, the usage of anti‐anxiety/depression medications, the presence of diabetic nephropathy, and cardiovascular disease.

## 4. Discussion

In this retrospective cohort study, we described the prevalence of adverse psychological states based on HADS and their association with ocular prognosis, including TRD involving the fovea, final BCVA <0.3, recurrent VH, and NVG, among VTDR patients. The results highlighted that increasing HADS scores and poorer psychological status, particularly depressive symptoms, were significantly associated with the higher risk of VTDR‐related poorer ocular prognosis.

As the major psychological risk factors, depression and anxiety were common in the patients with diabetes and its vascular complications. It is reported that about 28% of patients diagnosed with diabetes were suffering from depression [20] and similarly, the prevalence of anxiety was also up to 30% [21], where a mutually reinforcing relationship exists. The association between depression and DR was also revealed, with a higher incidence of depression increasing the risk of DR (OR = 1.35 to 2.46), but no significant association was observed between anxiety and DR, which was consistent with our findings [22]. Although few studies have specifically focused on the prevalence of depression and anxiety in patients with VTDR and their relationship with adverse ocular outcomes, the previous publications have indicated the potential positive correlation between DR severity and adverse psychological status [17, 23]. Our study, consistent with earlier reports, found that VTDR patients with higher HADS scores had a greater prevalence of adverse ocular outcomes. However, it must be acknowledged that while this association was consistent across the spectrum of scores, it should not be interpreted as evidence of causality, but rather as a correlational observation that warrants further systematic investigation.

Multiple mechanisms have been elucidated to explain how suboptimal mental states correlate with adverse outcomes in DR. Typically, DR progression stems from elevated inflammatory responses and subsequent neovascularization, potentially triggering local fibrotic tissue proliferation, TRD, and ultimately, poor visual prognosis [2]. Notably, emotional distress may contribute substantially to systemic homeostatic dysregulation. Studies have reported psycho‐physiological mechanisms underlying DR progression: increased systemic inflammation existed in individuals with diabetes, which was frequently exacerbated by comorbid depression. Significantly elevated levels of inflammatory cytokines have been documented in DM patients with depression, acting as a crucial mediating factor in the development of diabetes‐related microvascular complications. Conversely, antidepressant usage may demonstrably attenuate this positive association [24, 25]. Critically, adverse mental states also contribute to the development and progression of diabetic complications through their impact on daily behaviors, termed the psycho‐behavioral mechanism. For instance, individuals with anxiety or depression frequently exhibit unhealthy lifestyles, including higher rates of smoking, alcohol abuse, substance misuse, poor dietary habits, physical inactivity, and insufficient sleep, all recognized risk factors for DR progression [26]. Furthermore, delayed initiation and poor adherence to standardized treatments represent significant contributors to disease progression in DR patients with comorbid depression or anxiety. Depressed patients often experience severe social withdrawal and isolation, leading to diminished help‐seeking behavior, which substantially hinders engagement with necessary diagnostic and therapeutic interventions, accelerating DR development [27].

No significant association was observed between an elevated HADS‐A score and poor ocular prognosis, apart from worse final BCVA. Several factors explain this phenomenon. First, compared to depressed patients, those with anxiety typically exhibit less severe negative affect, which fundamentally attenuates their psychological and physiological pathological burden [28]. Moreover, anxious patients generally demonstrate higher self‐awareness, treatment adherence, and health care utilization, partially facilitating earlier intervention engagement [29, 30]. Effective control of these pathogenic factors consequently mitigates DR progression and reduces the incidence of sight‐threatening complications. Additionally, the median age (57 years) and diabetic duration (15 years) in the cohort may partially alleviate anxiety severity, as older age groups often develop enhanced coping mechanisms that attenuate anxiety responses, ultimately resulting in fewer adverse clinical outcomes [31].

Our study suggested that the high prevalence of anxiety and depression among patients with VTDR might be closely associated with adverse ocular outcomes, highlighting the necessity for early psychological screening and intervention in this population. Some psychological interventions, such as cognitive behavioral therapy (CBT) have demonstrated promising results. Qasir et al. [30] conducted a randomized controlled trial in type 2 diabetes patients, finding that CBT effectively alleviated stress, anxiety, and depressive symptoms, improved quality of life, and enhanced treatment adherence and lifestyle modifications. Similarly, it reduced illness‐related distress and suggested potential benefits for diabetic risk factor control in type 1 diabetes [32]. The beneficial effects have also been demonstrated in managing diabetic neurovascular complications, alleviating patient suffering, and improving their quality of life in diabetic peripheral neuropathy [33] and DN [34]. However, research specifically examining the impact of psychological interventions on ocular outcomes in DR patients remains limited. Gwyneth et al. implemented problem‐solving therapy in DR patients, demonstrating significant improvements in glycemic control (HbA1c levels) [35]. This finding suggests that active psychological intervention may improve the visual prognosis in VTDR patients.

Several limitations affect the generalizability of this study’s findings. First, the sample consisted exclusively of hospital‐recruited VTDR patients with complete medical records; therefore, undiagnosed or untreated DR patients whose psychological status had not been evaluated were not represented. Second, reliance on medical records and patient self‐report for key variables may have introduced measurement bias. Third, mental health status is context‐dependent. Although we adjusted for socioeconomic status, other diabetic complications and the usage of anxiolytic/antidepressant medications, and excluded participants with a history of severe psychological trauma, HADS scores can still be influenced by numerous factors, and a bidirectional relationship might exist. Of note, the retrospective design cannot fully capture the potential impact of subclinical psychiatric history without pharmacological treatment on baseline HADS scores. In addition, differences in medication type, dosage, and adherence were not analyzed, which represents an inherent limitation of this study. Future research incorporating structured psychiatric diagnostic interviews is warranted for further validation. Furthermore, improvements in metabolic control and lifestyle factors during the follow‐up period (up to 2 years) cannot be ignored, as they may partly affect our conclusions. Finally, the study was a retrospective observational design, and causality cannot be established. Well‐designed prospective cohort studies, investigations of underlying molecular mechanisms, and rigorous testing of the causal relationship between the psychological status and VTDR progression are warranted.

## 5. Conclusion

Collectively, this study demonstrates that elevated anxiety and depression scores, with particularly pronounced effects of depressive symptoms, are significantly associated with adverse ocular outcomes in patients with VTDR. Therefore, systematic psychological assessment and timely proactive intervention are important for mitigating the risk of vision deterioration among VTDR patients.

**Nomenclature**
ACR: Albumin to creatinine ratio
BCVA: Best‐corrected visual acuity
BMI: Body mass index
CBT: Cognitive behavioral therapy
CI: Confidence interval
CVD: Cardiovascular disease
DME: Diabetic macular edema
DN: Diabetic nephropathy
DR: Diabetic retinopathy
eGFR: Estimated glomerular filtration rate
ETDRS: Early treatment diabetic retinopathy study
HADS: Hospital anxiety and depression scale
HbA1c: Glycated hemoglobin
IQR: Interquartile range
LDL: Low‐density lipoprotein
NPDR: Nonproliferative DR
NVG: Neovascular glaucoma
OCT: Optical coherence tomography
OR: Odds ratio
PDR: Proliferative DR
PPV: Pars plana vitrectomy
PRP: Pan‐retinal photocoagulation
SBP: Systolic blood pressure
TRD: Tractional retinal detachment
VEGF: Vascular endothelial growth factor
VH: Vitreous hemorrhage
VTDR: Vision‐threatening DR.

## Author Contributions

Conceptualization: Bo Li, Chuandi Zhou and Zhi Zheng. Data collection, methodology and formal analysis: Bo Li and Chenxin Li. Writing – original draft: Bo Li, Yujin Jiang, and Yikeng Huang. Writing – review and editing: Shuzhi Zhao, Chuandi Zhou, Xiaofeng Zhang, and Zhi Zheng. Supervision and project administration: Zhi Zheng, Chuandi Zhou, and Xiaofeng Zhang. Funding acquisition: Bo Li, Zhi Zheng, Chuandi Zhou, and Xiaofeng Zhang.

## Funding

This work was supported by the Suzhou Health Talent Program (Grant SZWSRC2025081), the Suzhou Industrial Park Health Talent Support Program (Grant (2025) 31), the National Natural Science Foundation of China (Grants 82271111 and 82471104), the Clinical Research Innovation Plan of Shanghai General Hospital (Grant CTCCR‐2021C01), and the Suzhou Key Supporting Disciplines (Grant SZFCXK202123). The sponsor or funding organization had no role in the design or conduct of this research.

## Disclosure

All authors approved the final manuscript.

## Ethics Statement

This study was approved by the institutional review board, Shanghai General Hospital, Shanghai Jiao Tong University School of Medicine (identifier, 2022KY024). And the requirement for informed consent was waived owing to the retrospective design of the study. All the patients participated in this study voluntarily without additional compensation. The tenets of the Declaration of Helsinki were adhered to.

## Consent

The authors have nothing to report.

## Conflicts of Interest

The authors declare no conflicts of interest.

## Data Availability

The datasets used and/or analyzed during the current study are available from the corresponding author upon reasonable request.
